# Supplementary material for: Planar aggregation of the influenza viral fusion peptide alters membrane structure and hydration, promoting poration
Source: Nat Commun. 2022 Dec 5;13:7336. doi: 10.1038/s41467-022-34576-z (PMC9722698; doi:10.1038/s41467-022-34576-z)
Supplement: Supplementary file 2 — Description of Additional Supplementary Files [file 41467_2022_34576_MOESM2_ESM.pdf]

Description of Additional Supplementary Files

File Name: Supplementary Data 1

Description: Molecular dynamics initial and final coordinates in PDB format.

File Name: Supplementary Movie 1

Description: Final coordinates of the 1 FP in chol:POPC simulation. The simulation is oriented with the membrane normal "up", and in the video the view rotates twice (720°) about the membrane normal.

File Name: Supplementary Movie 2

Description: Final coordinates of the 1 FP in POPC simulation. The simulation is oriented with the membrane normal "up", and in the video the view rotates twice (720°) about the membrane normal.

File Name: Supplementary Movie 3

Description: Final coordinates of the 6 FP in chol:POPC simulation. The simulation is oriented with the membrane normal "up", and in the video the view rotates twice (720°) about the membrane normal.

File Name: Supplementary Movie 4

Description: Final coordinates of the 6 FP in POPC simulation. The simulation is oriented with the membrane normal "up", and in the video the view rotates twice (720°) about the membrane normal.

File Name: Supplementary Movie 5

Description: Final coordinates of the 10 FP in chol:POPC simulation. The simulation is oriented with the membrane normal "up", and in the video the view rotates twice (720°) about the membrane normal.

File Name: Supplementary Movie 6

Description: Final coordinates of the 10 FP in POPC simulation. The simulation is oriented with the membrane normal "up", and in the video the view rotates twice (720°) about the membrane normal.
