# Supplementary material for: Planar aggregation of the influenza viral fusion peptide alters membrane structure and hydration, promoting poration
Source: Nat Commun. 2022 Dec 5;13:7336. doi: 10.1038/s41467-022-34576-z (PMC9722698; doi:10.1038/s41467-022-34576-z)
Supplement: Supplementary file 5 — Reporting Summary [file 41467_2022_34576_MOESM5_ESM.pdf]

nature portfolio

Corresponding author(s): Joshua Zimmerberg

Last updated by author(s): 10/22/2022

Reporting Summary

Nature Portfolio wishes to improve the reproducibility of the work that we publish. This form provides structure for consistency and transparency in reporting. For further information on Nature Portfolio policies, see our Editorial Policies and the Editorial Policy Checklist.

Statistics

For all statistical analyses, confirm that the following items are present in the figure legend, table legend, main text, or Methods section.

n/a	Confirmed	
☐	☒	The exact sample size (*n*) for each experimental group/condition, given as a discrete number and unit of measurement
☐	☒	A statement on whether measurements were taken from distinct samples or whether the same sample was measured repeatedly
☐	☒	The statistical test(s) used AND whether they are one- or two-sided<br>*Only common tests should be described solely by name; describe more complex techniques in the Methods section.*
☒	☐	A description of all covariates tested
☒	☐	A description of any assumptions or corrections, such as tests of normality and adjustment for multiple comparisons
☐	☒	A full description of the statistical parameters including central tendency (e.g. means) or other basic estimates (e.g. regression coefficient) AND variation (e.g. standard deviation) or associated estimates of uncertainty (e.g. confidence intervals)
☐	☒	For null hypothesis testing, the test statistic (e.g. *F*, *t*, *r*) with confidence intervals, effect sizes, degrees of freedom and *P* value noted<br>*Give P values as exact values whenever suitable.*
☒	☐	For Bayesian analysis, information on the choice of priors and Markov chain Monte Carlo settings
☒	☐	For hierarchical and complex designs, identification of the appropriate level for tests and full reporting of outcomes
☒	☐	Estimates of effect sizes (e.g. Cohen's *d*, Pearson's *r*), indicating how they were calculated

Our web collection on statistics for biologists contains articles on many of the points above.

Software and code

Policy information about availability of computer code

Data collection: GUVs were imaged on a Zeiss LSM 880 microscope controlled by the Zen Black software. Microscopy images were analyzed using ImageJ (v1.53c NIH) implemented in FIJI v2.1.0. Simulations were performed using CHARMM (Chemistry at HARvard Macromolecular Mechanics) c41b1, OpenMM v7.4.1, Rickflow v0.7.0, and simulation code on the Anton 2 supercomputer (D. E. Shaw Research) v 1.56.0c7.

Data analysis: Experimental data were analyzed using SigmaPlot (Systat Software, Inc., Chicago, IL), Excel (v12.5 Microsoft, Inc., Redmond, WA), and MATLAB (v2018a-v2021a The MathWorks, Inc., Natick, MA). Simulation data were analyzed using VMD (Visual Molecular Dynamics) v 1.9.4a51, LOOS (Lightweight Object-Oriented Structure library) v3.3.0, the freud python library v2.11.0, MEMBPLUGIN v1.1, and cpptraj 18.

For manuscripts utilizing custom algorithms or software that are central to the research but not yet described in published literature, software must be made available to editors and reviewers. We strongly encourage code deposition in a community repository (e.g. GitHub). See the Nature Portfolio guidelines for submitting code & software for further information.

Data

Policy information about availability of data

All manuscripts must include a data availability statement. This statement should provide the following information, where applicable:

- Accession codes, unique identifiers, or web links for publicly available datasets
- A description of any restrictions on data availability
- For clinical datasets or third party data, please ensure that the statement adheres to our policy

All experimental data generated and analyzed in this article and its supplementary information files are available from the corresponding author upon request. The source data underlying Figures 1, 2, 4, 6, 7, 9 and Supplementary Figures 1-3, 5, 6, 8-11 are provided as a Source Data file. Initial and final coordinates of all Molecular Dynamics simulations are provided as Source Data in PDB format. All Molecular Dynamics trajectories generated on Anton 2 can be downloaded from the

Pittsburgh Supercomputer Center (PSC). The remaining Molecular Dynamics trajectories are available at the LoBoS cluster at NIH (contact R.W.P. at pastorr@nhlbi.nih.gov).

Field-specific reporting

Please select the one below that is the best fit for your research. If you are not sure, read the appropriate sections before making your selection.

☒ Life sciences ☐ Behavioural & social sciences ☐ Ecological, evolutionary & environmental sciences

For a reference copy of the document with all sections, see nature.com/documents/nr-reporting-summary-flat.pdf

Life sciences study design

All studies must disclose on these points even when the disclosure is negative.

Sample size	No sample size calculations were performed for this study. However, our previous work establishing the GUV poration assay used to determine leakage indicated that significant differences with adequate effect size were obtainable using ~50 – 100 GUV where square root N counting error is expected. The full kinetic time courses (Fig. 1B and poration time analysis) were difficult and challenging experiments using our flow chamber but the total reported (N=7 influx and N=2 efflux) led to consistent and significant distributional properties as indicated in the text "Specifically, poration occurs with a characteristic time, 101 +/- 22 sec, and normalized FP density, 2.7 +/- 0.05, (mean +/- SEM; n = 9 including both Alexa 488 and dextran experiments), both log-normally distributed, where the characteristic time represents the difference between the time leakage is first detected and the time the normalized FP fluorescence on the vesicles increases above the FP fluorescence in solution (see Fig. 9 and aggregation analyses in Methods)."
Data exclusions	No data was excluded.
Replication	For a given lipid composition, ~50 – 100 GUVs were observed and scored based on whether they have undergone influx of Alexa 488; 3 independent experiments were performed for two of the compositions and 2 for a third set of experiments. For the kinetic experiments, 9 GUV's from 5 different preparations were analyzed. For the dual dye uptake experiments, N=3 for both conditions. All attempts at replication were successful and confirmed by the resulting statistical properties of the data distributions (see Sample Size comments).
Randomization	No defined randomization scheme was established. However, experiments performed over the time of study (a potential covariate) were not done sequentially. For example, the different compositions probed varied over the course of the data collection period such that all experiments for any one condition did not occur at one specific time.
Blinding	Investigators were not blinded as they prepared, performed, and analyzed experiments because all preliminary analysis, preparations and experiments were performed primarily by one individual. Subsequent analysis by others used all the data eliminating bias by omission and the evaluation of statistical properties for comparisons also used all the data with our conclusions guided solely by the outcomes of any analysis and reported as such.

Reporting for specific materials, systems and methods

We require information from authors about some types of materials, experimental systems and methods used in many studies. Here, indicate whether each material, system or method listed is relevant to your study. If you are not sure if a list item applies to your research, read the appropriate section before selecting a response.

Materials & experimental systems

n/a	Involved in the study	
☒	☐	Antibodies
☒	☐	Eukaryotic cell lines
☒	☐	Palaeontology and archaeology
☒	☐	Animals and other organisms
☒	☐	Human research participants
☒	☐	Clinical data
☒	☐	Dual use research of concern

Methods

n/a	Involved in the study	
☒	☐	ChIP-seq
☒	☐	Flow cytometry
☒	☐	MRI-based neuroimaging
